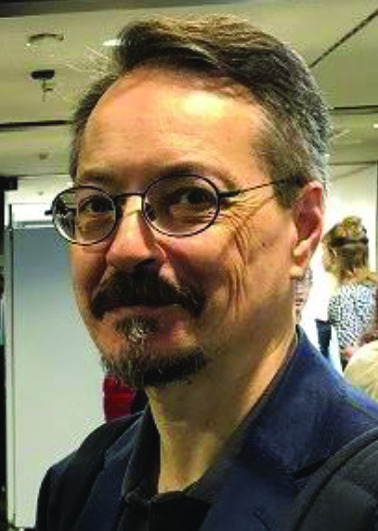# Sustainable Hydrogen Production by Glycerol and Monosaccharides Catalytic Acceptorless Dehydrogenation (AD) in Homogeneous Phase

**DOI:** 10.1002/cssc.202400639

**Published:** 2024-11-27

**Authors:** Sylwia Kostera, Luca Gonsalvi

**Affiliations:** ^1^ Istituto di Chimica dei Composti Organometallici (ICCOM) Consiglio Nazionale delle Ricerche (CNR) Via Madonna del Piano 10 50019 Sesto Fiorentino (Florence) Italy

**Keywords:** Acceptorless dehydrogenation, Green hydrogen production, Glycerol, Monosaccharides, Homogenous catalysis

## Abstract

In the quest for sustainable hydrogen production, the use of biomass‐derived feedstock is gaining importance. Acceptorless Dehydrogenation (AD) in the presence of efficient and selective catalysts has been explored worldwide as a suitable method to produce hydrogen from hydrogen‐rich simple organic molecules. Among these, glycerol and sugars have the advantage of being inexpensive, abundant, and obtainable from fatty acid basic hydrolysis (biodiesel industry) and from biomass by biochemical and thermochemical processing, respectively. Although heterogeneous catalysts are more widely used for hydrogen production from biomass‐based feedstock, the harsh reaction conditions often limit their applicability due to the deactivation of active sites caused by the coking of carbonaceous materials. Moreover, heterogeneous catalysts are more difficult to fine‐tune than homogeneous counterparts, and the latter also allow for high process selectivities under milder conditions. The present Concept article summarizes the main features of the most active homogeneous catalysts reported for glycerol and monosaccharides AD. In order to directly compare hydrogen production efficiencies, the choice of literature works was limited to reports where hydrogen was clearly quantified by yields and turnover numbers (TONs). The types of transition metals and ligands are discussed, together with a perspective view on future challenges of homogeneous AD reactions for practical applications.

## Graphical abstract

Sustainable hydrogen production can be obtained from hydogen‐rich biomass‐derived organic molecules through Acceptorless Dehydrogenation (AD) reactions. Glycerol and sugars are available in large quantities from industry and can be successfully used as substrates in AD protocols, in the presence of tailored, selective, easily tunable and thermally robust homogeneous catalysts. The main features of the most active homogeneous catalysts with quantified hydrogen production are hereby discussed, together with a perspective view on future challenges of homogeneous AD reactions for practical applications.

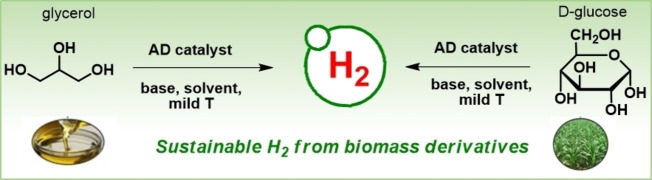

## Introduction

The current global utilization of fossil‐derived feedstocks and endangered, rare raw materials for energy, transportation and in the fine chemicals industry, is threatening for the future economic and environmental sustainability, and in turn large quantities of greenhouse gases are produced from these sources and processes.^[1]^ The 2015 Paris Agreement proposed measures to curb global climate change, setting the 20–20‐20 targets by 2020 (20 % increase in energy efficiency, 20 % reduction of CO_2_ emissions, and 20 % use of renewables).[^2^, ^3^] The 2016 European Commission Clean Mobility Package outlined the transition to low‐ or zero‐emission mobility, targeting a 60 % decrease of greenhouse gas emissions by 2050^[4]^ A sustainable approach to energy, fuels and chemical industry must rely on the use of cheap and abundant raw materials, new technologies and solutions for the efficient use of waste, to foster Reduce‐Reuse‐Recycle (3R) within Circular Economy. Biomass from different sources, including agricultural waste, has been identified as a sustainable feedstock for the production of biofuels, platform chemicals and value‐added products. The production of renewable chemicals from biorefineries is expected to grow in the coming years with an increased market share.^[5]^

Bioalcohols, polyols and sugars derived from biomass are receiving increased attention as sustainable raw materials to be used as platform chemicals. Ethanol (CH_3_CH_2_OH) is one of the most promising biofuels, frequently used in blended gasoline in the concentration range 10–85 % (v/v). Apart from the direct use, biomass derivatives can in principle be exploited as feedstock for energy production.

**Figure 1:**
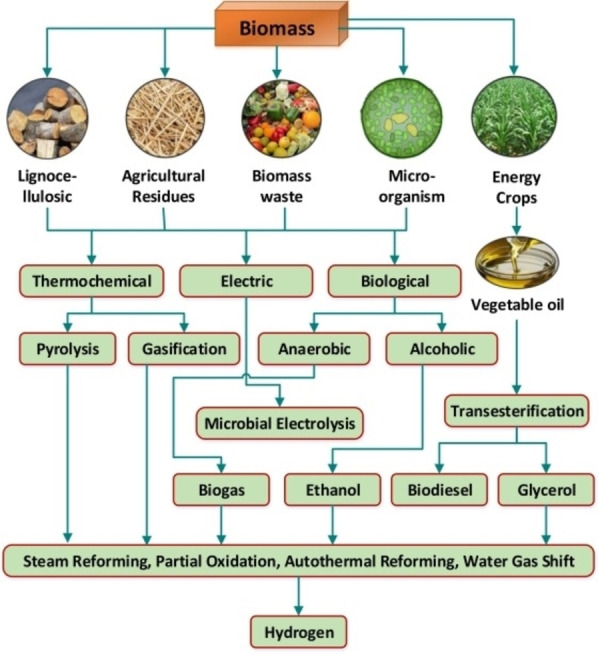
Hydrogen production processes from biomass using different methods. Reprinted from^[6]^ with permission from Elsevier.

In the growing field of research on sustainable energy vectors, hydrogen (H_2_) is receiving renewed interest, as it can be converted to electricity using the by‐now mature fuel cell technology. Various alternative approaches to the current use of steam reforming of fossil fuels are currently considered for the generation of hydrogen,^[6]^ the most studied of which is water electrolysis, that can benefit from the growing body of research and technology breakthroughs in the sustainable electricity production from solar and wind power. The use of biomass derivatives for sustainable hydrogen production^[7]^ is a field of research and applications in rapid growth, reaching different technology readiness levels (TRLs) up to lab‐to‐market applications.^[8]^ These processes are commonly classified as thermochemical (gasification, pyrolysis, aqueous phase reforming)[^9^, ^10^] and biological approaches (biological water gas shift reaction, photo‐fermentation and dark fermentation). All these methods however suffer from different drawbacks, such as high energy request, poor selectivity and low hydrogen yields. A visual representation of the principal biomass sources and processes to obtain hydrogen is shown in Figure 1.

**Scheme 1:**
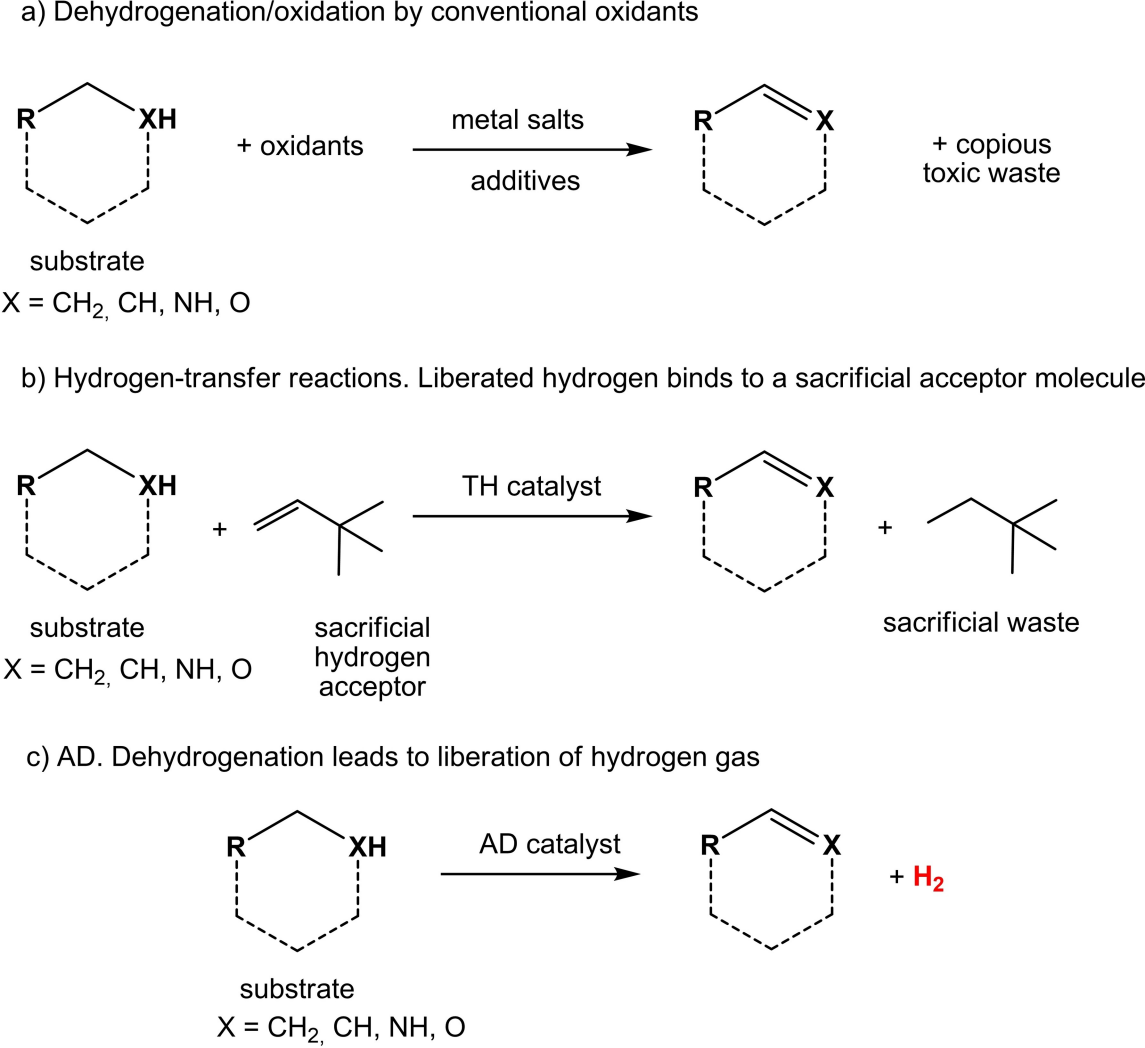
General scheme of dehydrogenation reactions, showing the advantage of AD protocols over conventional reactions.^[14]^

An alternative way to produce H_2_ is to extract it from renewable, stable, cheap and abundant hydrogen‐rich simple organic molecules, including alcohols derived from biomass (methanol, ethanol and polyols) using efficient and selective catalytic dehydrogenation reactions.[^11^, ^12^] Among possible substrates for dehydrogenation of biomass‐derived products, monosaccharides (among which glucose, fructose, xylose, sorbitol are the most represented) and glycerol, obtained as by‐product from vegetable fatty esters processing, showed promising results by heterogeneous catalytic processes.^[13]^ In the case of use of alcohols as substrates, and in the absence of H‐acceptor unsaturated molecules, this type of reaction is conventionally named *Acceptorless Alcohol Dehydrogenation* (AAD). AAD can be considered as a green and sustainable alternative to classical oxidation chemistry, as it does not require the use of conventional oxidants or sacrificial acceptors, and in turn does not produce stoichiometric amounts of waste. Another advantage is that the formation of oxidized product(s) is accompanied by formation of gaseous H_2_ that can be either used as an energy source or as a reductant in tandem reactions (Scheme 1).^[14]^ For this reason, AAD protocols have been traditionally applied as synthetic methods for the conversion of alcohols to ketones,[^15^, ^16^] for the coupling alcohols with various nucleophiles via the formation of C−O, C−N, C−S, C−C, and C=C bonds,^[17]^ and for demanding C−H activation of alkanes to alkenes.^[18]^

Whereas the use of heterogeneous catalysts for AAD has been widely reported,[^19^, ^20^, ^21^] these protocols generally need high temperatures to achieve high productivities, and may suffer from low selectivity towards hydrogen product. Another major disadvantage is limited catalyst durability due to carbonaceous material deposition (cooking) on the catalyst surface, that may deactivate the active metal sites, in turns requiring tedious, energy‐intensive and costly catalyst regeneration. A possible alternative, that is gaining importance,^[22]^ is the use of homogeneous catalysis relying on transition metal complexes bearing functional ligands. Transition metals have incompletely filled d‐orbitals available to both donate and accept electrons easily from substrates to be converted. In the case of hydrogenation/dehydrogenation cycles, many precious and non‐precious transition metals are able to bring about hydride abstraction from hydrogen‐rich molecules, and in the presence of a proton source, eliminate H_2_. This may occur by inner‐sphere oxidative addition/reductive elimination steps (requiring 2‐electrons oxidation state changes on the metal) or by outer‐sphere cooperative activation. In the latter case, the metal does not need to change its oxidation state, and the mechanism relies on the presence of Lewis base functional site on the stabilizing ligand to construct an intra‐molecular Lewis acid‐base pair with the metal, as in the case of Noyori‐type hydrogenation catalysis.^[23]^ These concepts have been recently discussed in an excellent review article.^[24]^ Well‐defined transition metal catalysts also have the advantage over heterogeneous ones of a higher synthetic modularity that allows fine‐tuning of the electronic and steric properties around the active metal centers. Drawbacks may be a limited catalyst lifetime and a poor recyclability, that need to be overcome by further research and catalyst optimization. Examples of homogeneous AAD of biomass‐based substrates have been recently summarized,^[11]^ and previously for the production of lactic acid (and hydrogen) from glycerol,^[25]^ but in general the majority of reports targets the synthesis of added‐value organic products rather than hydrogen. In this Concept article, the latest results on hydrogen production by glycerol and monosaccharides by *Acceptorless Dehydrogenation* (AD) reactions from literature data that report measured hydrogen production will be summarized, comparing different type of catalysts, the choice of metal and ligands, hydrogen productivities (TONs) and reaction conditions.

### Hydrogen Production by Homogeneous Catalytic AD of Glycerol

**Scheme 2:**
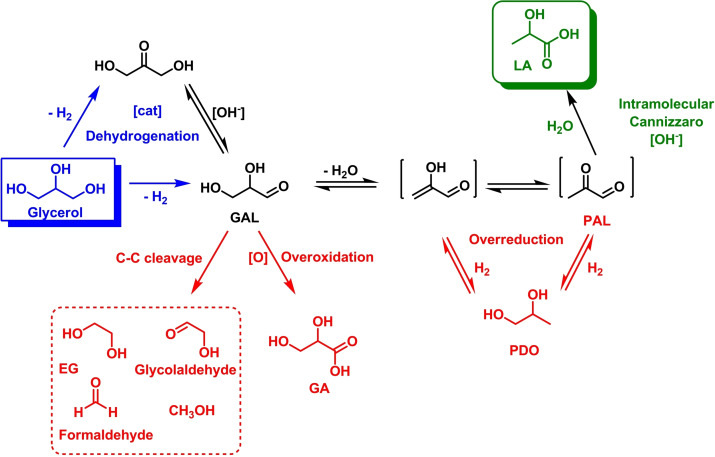
Reactions network and product distribution that can be obtained starting from glycerol catalytic dehydrogenation.^[41]^

**Figure 2:**
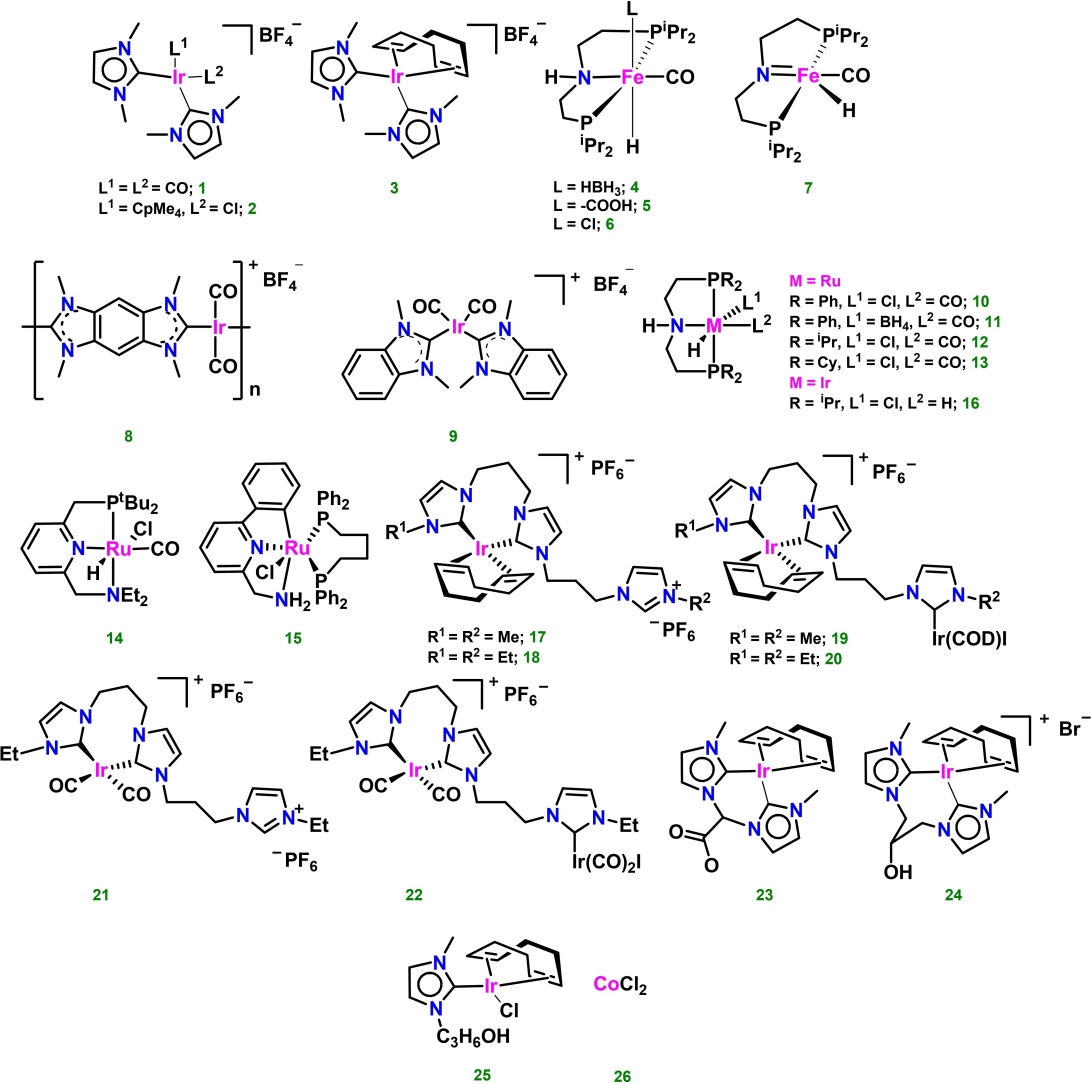
Fe, Ru and Ir complexes used as homogenous catalysts in glycerol dehydrogenation. Complexes **10**–**14** and **16** were also applied for monosaccharides dehydrogenation (vide infra).

In this section, hydrogen productivity of the most active homogeneous systems for glycerol AD will be compared. The molecular catalysts described in the literature are based mainly on iridium, ruthenium and cobalt complexes. Glycerol is a widely studied substrate also due to its use as a model system for the production of H_2_ from other polyols or more complex biomass. In the majority of the publications, the authors focused on the conversion of glycerol to products such as lactic acid, 1,3‐dihydroxypropan‐2‐one and/or other polyols. In some cases hydrogen was detected, but no quantitative information was provided.[^26^, ^27^, ^28^, ^29^, ^30^] Scheme 2 shows the reactions network of glycerol dehydrogenation and the possible product distribution. The complexity and number of the possible side reactions that may be triggered upon glycerol dehydrogenation is a serious issue when targeting a specific product, that makes the choice of highly selective catalysts extremely important. For homogeneous glycerol AD reactions, previous research on ethanol and methanol AAD have inspired researchers to develop catalysts based on metals such as Ir, Ru, and more recently Fe, principally for the conversion of glycerol to lactic acid under mild conditions.[^31^, ^32^, ^33^] The design of ligands and catalysts plays a crucial role in creating efficient, rapid, and robust homogeneous catalysts using precious and earth‐abundant metals. In the choice of stabilizing ligands, transition metal catalysts incorporating pincer‐type ligands have exhibited impressive efficiencies in the dehydrogenation of substrates such as formic acid,^[34]^ primary and secondary alcohols,^[35]^ methanol^[36]^ and nitrogen‐containing heterocycles.^[37]^ Pincer ligands force rigid meridional geometries around the metal center, stabilize metal low oxidation states, have high electron donor abilities and are generally easily synthetically tunable to get desired steric and electronic properties. Importantly, some of them can act as non‐innocent ligands triggering Noyori‐type metal‐ligand cooperation (MLC) mechanisms.[^38^, ^39^] Another class of ligands able to coordinate transition metals by strong M−C bonds is represented by *N*‐heterocyclic carbenes (NHCs). Moreover, bis‐NHC ligands enhance further the stability of organometallic complexes due to chelation, making them highly resistant even under harsh reaction conditions. Hemilabile carboxylate or hydroxyl groups in the NHCs backbone give higher solubility and stability in polar solvents, often used for glycerol AD.^[40]^ Molecular drawings of some of the most efficient catalysts described for H_2_ production from glycerol AD are shown in Figure 2. A comparison of TON values and reaction conditions is summarized in Table 1.

**Table 1:** Hydrogen production from acceptorless glycerol dehydrogenation in homogeneous phase.

Entry	Catalyst	Cat. amount [% mol]	Base	Solvent	Temperature [°C]	Time [h]	TON^[a]^	Refs.
1	**1**	0.007	KOH	–	115	15	4500	[41]
2	**2**	0.007	KOH	–	115	15	750	[41]
3	**3**	0.007	KOH	–	115	15	2500	[41]
4	**4**	0.02	NaOH	H_2_O/NMP	140	3	525	[42]
5	**5**	0.02	NaOH	H_2_O/NMP	140	3	700	[42]
6	**6**	0.02	NaOH	H_2_O/NMP	140	3	600	[42]
7	**7**	0.02	NaOH	H_2_O/NMP	140	3	300	[42]
8	**8**	0.3	KOH	H_2_O	165	48	2920^[b]^	[43]
9	**9**	0.3	KOH	H_2_O	165	48	1927^[b]^	[43]
10	**10**	0.001	KOH	Diglyme	125	2	5914^[c]^	[44]
11	**11**	0.003	NaOH	Diglyme	125	2	1722^[c]^	[44]
12	**12**	0.003	NaOH	Diglyme	125	2	1418^[c]^	[44]
13	**13**	0.003	NaOH	Diglyme	125	2	1372^[c]^	[44]
14	**14**	0.003	NaOH	Diglyme	125	2	1162^[c]^	[44]
15	**15**	0.003	NaOH	Diglyme	125	2	466^[c]^	[44]
16	**16**	0.003	NaOH	Diglyme	125	2	1132^[c]^	[44]
17^[d]^	**10** ^[b]^	0.0001	KOH	Diglyme	125	2	20636^[c]^	[44]
18^[d]^	**10** ^[b,c]^	0.0001	KOH	Diglyme	125	2	50176^[c]^	[44]
19^[e]^	**10** ^[b,d]^	0.0001	KOH	Diglyme	125	2	29668^[c]^	[44]
20	**17**	0.0007	Ba(OH)_2_	H_2_O	180	7.5	104250^[c]^	[45]
21	**18**	0.0007	Ba(OH)_2_	H_2_O	180	7.5	63750^[c]^	[45]
22	**19**	0.0004	Ba(OH)_2_	H_2_O	180	7.5	59250^[c]^	[45]
23	**20**	0.0004	Ba(OH)_2_	H_2_O	180	7.5	81825^[c]^	[45]
24	**21**	0.0007	Ba(OH)_2_	H_2_O	180	7.5	5595^[c]^	[45]
25	**22**	0.0004	Ba(OH)_2_	H_2_O	180	7.5	26475^[c]^	[45]
26	**23**	0.20	KOH	–	130	5	179	[46]
27	**24**	0.20	KOH	–	130	5	150	[46]
28	**25**	0.20	KOH	–	130	5	146	[46]
29	**26**	0.75	KOH	EtOH	160	48	413^[b]^	[47]

[a] TON=(mmol H_2_ product)/(mmol catalyst). Mmol H_2_ calculated from Ideal Gas Law from volumes observed from gas burettes or from GC traces of sample gas mixtures. [b] Mmol H_2_ calculated from (H_2_ yield%)x(mmol of substrate)/100. [c] TON=(reported TOF, h^−1^)x(reported total reaction time, h). Reported as an average of 2 reactions and with an error margin of 10 %. [d] Industrial glycerol was used. [e] Mixture of glycerol and H_2_O, 86.5 % glycerol, 13.5 % H_2_O.

**Scheme 3:**
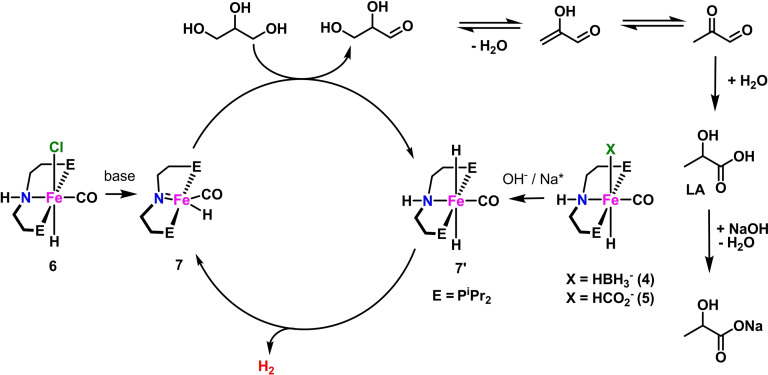
Proposed mechanism for glycerol AD in the presence of Fe pincer complexes **4**–**7**.^[42]^

Sharninghausen, Campos and coworkers reported on the use of Ir‐NHC type complexes **1**–**3** (Figure 2) as the first homogeneous catalysts for the dehydrogenation of glycerol to lactic acid and hydrogen. Complex **1** showed the highest activity for hydrogen production with TON=4500 (Table 1, entry 1), while complex **3** showed the lowest activity with TON=750 (Table 1, entry 2).^[41]^ Crabtree, Hazari and coworkers described glycerol dehydrogenation in the presence of Fe‐PNP pincer complexes **4**–**7**, targeting lactic acid (LA) as the desired product. For the most active system, hydrogen evolution was also quantified by volumetric analysis (gas burette). The tests were carried out at 140 °C using 0.02 mol % of the complex, 1 eq. NaOH vs. glycerol and a mixture of N‐methyl‐2‐pyrrolidinone (NMP)/water (1:1) as the solvent. The reactions were monitored for 3 h (Table 1, entries 4–7), and after 30 min a decrease in the rate of hydrogen generation was observed. Complex **5** showed the best activity (TON=700), whereas complexes **4**, **6** and **7** gave lower TON values of 525, 600 and 300, respectively.^[42]^ Tu and coworkers disclosed the use of other Ir‐NHC type catalysts, namely the monometallic complex **9** and the robust coordination polymer **8**, based on the rigid bis‐benzimidazolium salt, reaching TONs of 1927 and 2920, respectively (Table 1, entries 8 and 9). The reactions were carried out in an aqueous medium in the presence of KOH as base and at a temperature of 165 °C. Increasing the reaction temperature from 165 °C to 185 °C did not increase TON significantly, rather allowed for shorter reaction times.^[43]^ Beller and coworkers applied a small library of ruthenium and iridium complexes (Figure 2, complexes **10**–**16**) as catalysts for glycerol AD. The best results were obtained using the pincer type catalyst Ru‐MACHO (**10**) in the presence of KOH (Table 1, entry 10). A TON of 5914 was observed in diglyme at 125 °C. Lowering the loading of catalyst **10** led to a TON of 20636. To investigate the effect of the type of pincer ligand, other ruthenium complexes were tested (Table 1, entries 11–14). The reaction was carried out in the presence of NaOH. Both the change of the anionic substituent from Cl^−^ to BH_4_^−^ and the change of substituents on the phosphorus atom (Table 1, entry 11) resulted in a decrease in hydrogen production in the described reaction. The same occurred when complexes **14**, **15** and **16** were used. In addition, the authors tested industrial glycerol obtained as waste byproduct from biodiesel production (glycerol content: 86.5 %) and a mixture of glycerol and water (86.5 /13.5 %). Unexpectedly, the best results were obtained using industrial glycerol, obtaining a TON of 50176. The authors suggest that this may be due to increased base solubility. For the same reason, good results were also obtained using a glycerol/water mixture (TON=29668). The amount of gas generated over time was measured by a gas burette after removing blank volumes.^[44]^ An example of proposed mechanism for glycerol AD in the presence of pincer complexes was described by Crabtree and Hazari, namely for Fe‐PNP compounds **4**–**7** (Scheme 3).^[42]^

Complexes **4** and **5** act as precatalysts for the bis(hydride) complex **7′**, either through base (OH^−^) assisted loss of BH_3_ from **4** or Lewis acid (Na^+^) assisted decarboxylation of **5**. The amido complex **7** is instead obtained from **6** through deprotonation of the amine PNP moiety. Within the catalytic cycle, glycerol is dehydrogenated by **7** giving **7′** with reprotonation of the PNP ligand and hydride transfer to Fe. Next, **7′** releases hydrogen giving back **7** and closing the catalytic cycle. The product of glycerol dehydrogenation (glyceraldehyde) follows a series of base‐catalyzed, metal free reactions, namely dehydration, tautomerization and an intramolecular Cannizzaro reaction, to form lactic acid (LA). The mechanism shows the importance of the non‐innocent PNP pincer ligand in assisting substrate activation and H_2_ elimination through a MLC mechanism. Jang and coworkers investigated the use of monometallic and bimetallic iridium complexes stabilized by potentially tridentate NHC ligands (Scheme 2, complexes **17**–**22**) for hydrogen generation from glycerol. The reactions were carried out in the presence of barium hydroxide, in aqueous medium at 180 °C. Complexes **17** and **20** proved to be the most effective with TON of 104250 and 81825, respectively (Table 1, entries 20 and 23). The remaining complexes showed slightly lower efficiency for hydrogen generation (Table 1, entries 21, 22 and 25), the lowest been shown by **21** (Table 1, entry 27). Due to the unique structural features of linear tris(carbene) ligands, which can induce polymetallic coordination, it was concluded that the reaction rate of glycerol dehydrogenation increases due to the cooperative actions of metal ions.^[45]^ Pérez‐Torrente, Jiménez and coworkers reported on the dehydrogenation of net glycerol using KOH as base at 130 °C and iridium(I) compounds **23**–**25** as catalyst, featuring methylene or propylene‐bridged bis‐NHC ligands (loading 0.2 mol %). Acetate and hydroxy groups were placed as substituents on the bridge moieties to increase solubility in the reaction medium. Good conversions of hydrogen were observed after 5 h, corresponding to TONs of 179 (conversion of 98 %), 150 (conversion of 84 %) and 146 (conversion of 81 %) for the three catalyts, respectively (Table 1, entries 26–28). H_2_ evolution was monitored in a closed microreactor equipped with a pressure transducer and it was measured until constant pressure was reached. The amount of hydrogen (mmol) produced was calculated by the Ideal Gas Law.^[46]^ Kumar and coworkers presented acceptorless dehydrogenation of glycerol catalyzed by CoCl_2_ (**26**). The reaction was carried out in the presence of a base (KOH) in ethanol and at 160 °C. As a result, in addition to lactate, ethylene glycol and formate, hydrogen was obtained with a TON of 413 (Table 1, entry 29). Different loadings of anhydrous CoCl_2_ were tested, but the best result for H_2_ production was obtained applying 0.75 mol % of catalyst. Although showing low activity, the advantage of catalyst **26** resides its easy availability, low price, environmental friendliness and the use of an earth‐abundant metal.^[47]^

In summary, the best catalyst for the homogeneous glycerol AD is so far iridium complex **17** studied by Jang and coworkers, reaching TON=104250. The presence of a dangling NHC arm in the ligand system may favor activity by increasing catalyst solubility in the highly polar media used for glycerol dehydrogenation. Further research is needed to pinpoint the mechanistic details of the reaction with this type of Ir‐NHC catalysts. The next challenge for glycerol AD is to replace costly Ru and Ir complexes with efficient non‐noble metal counterparts. This approach has been already applied to the hydrogenation and dehydrogenation of organic substrates[^48^, ^49^, ^50^, ^51^, ^52^, ^53^, ^54^, ^55^, ^56^] and, in the case of glycerol AD, Fe(PNP) complexes showed H_2_ production with promising TON values (400–700). Based on these results, we believe that the study of novel and efficient earth‐abundant metal catalysts bearing functional ligands able to promote MLC pathways should be considered in the future to achieve more sustainable glycerol AD protocols.

### Hydrogen Production by Homogeneous Catalytic AD of Monosaccharides

**Scheme 4:**
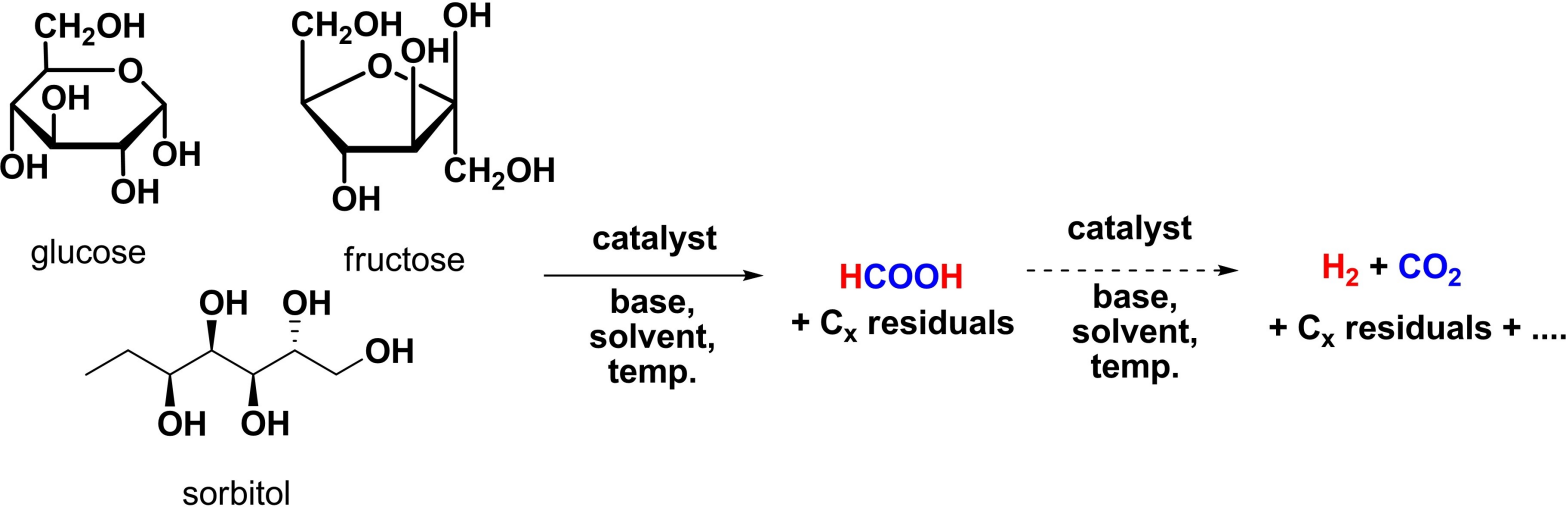
General reaction and products of monosaccharides catalytic dehydrogenation.

**Figure 3:**
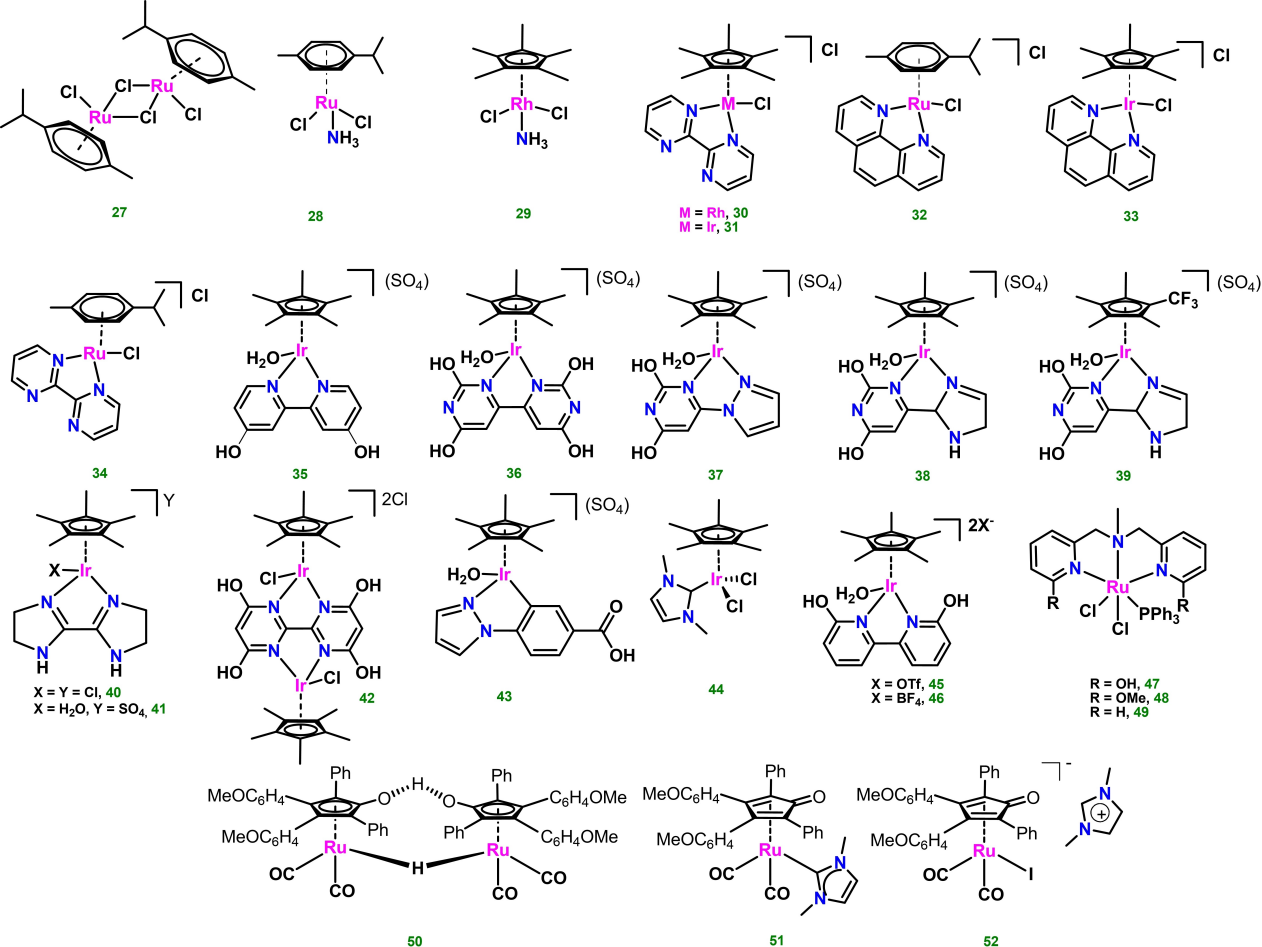
Ru, Rh and Ir complexes used as homogenous catalysts for monosaccharides acceptorless dehydrogenation.

This section summarizes the use of monosaccharides such as glucose, fructose, sorbitol etc. as substrates for AD reactions (Scheme 4). Drawings of the catalysts are shown in Figure 3.

In the quest for cheap and abundant feedstocks for hydrogen production, attention has been dedicated to non‐edible biomass and derivatives. Today, thermal and biological processes are commonly used, albeit under harsh dehydrogenation conditions requiring high energy input and costs. Agricultural or forestry residues are treated by thermal gasification (700–1400 °C), pyrolysis (300–1000 °C) or supercritical water gasification (>374 °C, 217 atm). These processes are characterized by low heat efficiency, high equipment cost and the need for downstream gas mixtures purification that limit the applicability of these technologies. By pre‐treatment of raw biomass such as wheat, corn and rice straw, typically composed of cellulose and hemicellulose, sugars such as glucose can be obtained in various amounts depending on the feedstock, and in turn these can be exploited as simple dehydrogenation substrates. In the reports on the use of sugars as substrates for AD reactions, the authors generally focused on the quantification of organic added‐value products such as gluconic acid[^57^, ^58^] or lactones.[^59^, ^60^] Detection of hydrogen was sometimes reported, but no information was provided on quantitative gas yields. In this section, the literature data reporting measured hydrogen production will be compared, as summarized in Table 2.

**Table 2:** Hydrogen production from acceptorless monosaccharides dehydrogenation in homogeneous phase.

Entry	Substrate	Catalyst	Cat. amount [% mol]	Base	Solvent	Temperature [°C]	Time [h]	TON^[a]^	Refs.
1	C_6_H_12_O_6_⋅H_2_O	**27**	0.44	TMEDA	IL1	150	1	58	[61]
2	C_6_H_12_O_6_⋅H_2_O	**27**	0.44	TMEDA	IL2	180	1	35	[61]
3	C_6_H_12_O_6_⋅H_2_O	**27**	0.44	TMEDA	IL3	180	1	31	[61]
4	C_6_H_12_O_6_⋅H_2_O	**27**	0.44	TMEDA	IL4	150	1	72	[61]
5	C_6_H_12_O_6_⋅H_2_O	**27**	0.44	TMEDA	IL5	180	1	11	[61]
6	L‐(−)‐fructose	**10**	0.005	KOH	Diglyme	95	2	1134^[b]^	[62]
7	L‐(−)‐fructose	**11**	0.005	KOH	Diglyme	95	2	1132^[b]^	[62]
8	L‐(−)‐fructose	**12**	0.005	KOH	Diglyme	95	2	2106^[b]^	[62]
9	L‐(−)‐fructose	**13**	0.005	KOH	Diglyme	95	2	1730^[b]^	[62]
10	L‐(−)‐fructose	**14**	0.005	KOH	Diglyme	95	2	546^[b]^	[62]
11	L‐(−)‐fructose	**16**	0.001	NaOH	Diglyme	95	6	11371^[b]^	[62]
12	D‐(+)‐glucose	**16**	0.001	NaOH	Diglyme	95	6	12177^[b]^	[62]
13	C_6_H_12_O_6_⋅H_2_O	**28**	0.02	–	H_2_SO_4(aq)_	98	1	270^[b]^	[63]
14	C_6_H_12_O_6_⋅H_2_O	**29**	0.02	–	H_2_SO_4(aq)_	98	1	72^[b]^	[63]
15	C_6_H_12_O_6_⋅H_2_O	**30**	0.01	–	H_2_SO_4(aq)_	98	1	7^[b]^	[63]
16	C_6_H_12_O_6_⋅H_2_O	**31**	0.01	–	H_2_SO_4(aq)_	98	1	<1^[b]^	[63]
17	C_6_H_12_O_6_⋅H_2_O	**32**	0.01	–	H_2_SO_4(aq)_	98	1	<1^[b]^	[63]
18	C_6_H_12_O_6_⋅H_2_O	**33**	0.01	–	H_2_SO_4(aq)_	98	1	<1^[b]^	[63]
19	C_6_H_12_O_6_⋅H_2_O	**34**	0.01	–	H_2_SO_4(aq)_	98	1	<1^[b]^	[63]
20	Wheat straw^[c]^	**16**	20.01	NaOH	H_2_SO_4(aq)_/DMSO	90	50	1103	[64]
21	Wheat straw^[c]^	**35**	20.01	NaOH	H_2_SO_4(aq)_/DMSO	90	4	3756	[64]
22	Wheat straw^[c]^	**36**	20.01	NaOH	H_2_SO_4(aq)_/DMSO	90	4	1627	[64]
23	Wheat straw^[c]^	**37**	20.01	NaOH	H_2_SO_4(aq)_/DMSO	90	1	4010	[64]
24	Wheat straw^[c]^	**38**	20.01	NaOH	H_2_SO_4(aq)_/DMSO	90	<1	4171	[64]
25	Wheat straw^[c]^	**39**	20.01	NaOH	H_2_SO_4(aq)_/DMSO	90	2	3607	[64]
26	Wheat straw^[c]^	**40**	20.01	NaOH	H_2_SO_4(aq)_/DMSO	90	4	1593	[64]
27	Wheat straw^[c]^	**41**	20.01	NaOH	H_2_SO_4(aq)_/DMSO	90	4	1485	[64]
28	Wheat straw^[c]^	**42**	20.01	NaOH	H_2_SO_4(aq)_/DMSO	90	3	3935	[64]
29	Wheat straw^[c]^	**43**	20.01	NaOH	H_2_SO_4(aq)_/DMSO	90	2	638	[64]
30	Wheat straw^[c]^	**38**	6.94	NaOH	H_2_SO_4(aq)_/DMSO	90	<0.5	13627	[64]
31	Wheat straw^[c]^	**38**	4.17	NaOH	H_2_SO_4(aq)_/DMSO	90	1	21943	[64]
32	D‐(+)‐glucose	**44**	2.00	–	H_2_SO_4(aq)_	100	20	49^[d]^	[70]
33	D‐(+)‐glucose	**45**	0.20	–	H_2_O	100	20	380 ^[d]^	[71]
34	D‐(+)‐glucose	**45**	1.00	–	H_2_O	100	20	95^[d]^	[71]
35	D‐(+)‐glucose	**46**	0.20	–	H_2_O	100	20	355^[d]^	[71]
36	Sorbitol	**10**	1.59	KOH	Diglyme/H_2_O	125	1	1025	[44]
37	Sorbitol	**47**	0.01	KOH	Diglyme/H_2_O	150	24	35359	[73]
38	Sorbitol	**48**	1.00	KOH	Diglyme/H_2_O	150	24	200^[e]^	[73]
39	Sorbitol	**49**	1.00	KOH	Diglyme/H_2_O	150	24	320^[e]^	[73]
40	Glucose	**50**	2.00	–	DMSO/H_2_O	150	4	104^[d]^	[74]
41	Glucose	**51**	2.00	–	DMSO/H_2_O	150	4	1^[d]^	[74]
42	Glucose	**52**	2.00	–	DMSO/H_2_O	150	4	3^[d]^	[74]
43	Fructose	**50**	2.00	–	DMSO/H_2_O	150	4	<1^[d]^	[74]
44	Arabinose	**50**	2.00	–	DMSO/H_2_O	150	4	<1^[d]^	[74]
45	Sorbitol	**50**	2.00	–	DMSO/H_2_O	150	4	8^[d]^	[74]

[a] TON=(mmol H_2_ product)/(mmol catalyst). Mmol H_2_ calculated from Ideal Gas Law from volumes observed from gas burettes or from GC traces of sample gas mixtures. [b] TON=(reported TOF, h^−1^)x(reported total reaction time, h). [c] Components of wheat straw: H_2_O (2.3 %), cellulose (41.2 %), hemicellulose (16.2 %), lignin (18.3 %), ash (5.5 %), extraction (13.4 %); carbon content 43.84 %, ca. 18 mmol based on cellulose and hemicellulose, [C_x_H(_2x−2_)O(_x−1_)]_n_ x=5, 6). Catalyst loading and H_2_ yields based on the carbon content. Wheat straw (0.94 g, 18.00 mmol C‐atoms of polysaccharides) used as starting material. Reaction conditions, first step: 0.7 wt % H_2_SO_4_ aqueous solution (30.00 ml), DMSO (0.31 ml, 1 v%), NaVO_3_ (81.3 mg, 4 mol %), air (3.0 bar), 160 °C, 3 h. Reaction conditions, second step: HCOOH (calculated as ca. 18 mmol), NaOH (10.0 M, 0.43 ml) added to neutralize H_2_SO_4_, [Ir] (3.75 mmol). The produced gas was identified by gas chromatography. [d] Mmol H_2_ calculated from (H_2_ yield%)x(mmol of substrate)/100. [e] Mmol H_2_ calculated from (H_2_ yield as equiv. to sorbitol)x(mmol of sorbitol feed).

Among the first reports on the use of homogeneous catalytic systems, Wasserscheid and coworkers presented a new ionic liquid‐based catalytic system that allows the production of hydrogen from glucose. The system was based on the use pf ionic liquids ([EMIM][Me‐P(OMe)O_2_] (**IL1**), [EMIM][H‐P(OMe)O_2_] (**IL2**), [EMIM][acetate] (**IL3**), [Bu_4_P][Me‐P(OH)O_2_] (**IL4**), [MMIM][Me_2_PO_4_] (**IL5**)) together with the commercially available ruthenium catalyst [RuCl_2_(p‐cymene)]_2_ (Scheme 4, complex **27**). The results of the AD tests are shown in Table 2, entries 1–5. The ionic liquids helped to dissolve the carbohydrate feedstock, dissolve and stabilize the catalyst and increase hydrogen solubility. The best results were obtained using ionic liquid **IL4** at 150 °C, obtaining hydrogen with a yield of 5.2 %. The ionic liquid/catalyst system demonstrated stability and durability to 48 h at 180 °C, allowing sixconsecutive glucose dehydrogenation reactions without formation of solid tarry materials. The amount of generated hydrogen was determined by integrating the detector signal *vs*. time. The gas phase and condensed liquid phases products were analyzed by GC‐MS. Isotopic labeling studies showed that glucose thermally decomposes in the applied ionic liquids through a dehydration/rehydration process, yielding one mol of formic acid per mol of glucose. HCOOH then undergoes selective Ru‐catalyzed dehydrogenation to H_2_ and CO_2_.^[61]^ Beller and coworkers disclosed results on the production of hydrogen from monosaccharides such as fructose and glucose in the presence of ruthenium complexes **10**–**14** and iridium complex **16** (Figure 2) under homogeneous conditions (Table 2, entries 6–12). The reactions were carried out under mild conditions at 95 °C using diglyme as solvent in the presence of 1.3 equiv. of KOH. This choice of solvent and base was suitable for hydrophilic sugars and compatible with sensitive catalysts. Complex **12** showed a slightly better activity using KOH in the presence of fructose as substrate, with a TON of 2106 (Table 2, entry 8). Catalyst **16** displayed very high activity using NaOH at low catalyst loadings from L‐(−)‐fructose with TON=11371 and from D‐(+)‐glucose with TON=12177 after 6 h (Table 2, entries 11 and 12). The reactions were monitored over time and the amount of generated gas was measured by a manual gas burette, whereas the gas purity was established by GC analysis.^[62]^

A family of water‐soluble ruthenium, iridium or rhodium organometallic complexes were tested for glucose dehydrogenation by Zhou and co‐workers (Figure 3, complexes **28**–**34**, entries 13–19). The authors described an unprecedented mild reaction system for hydrogen production from glucose using various complexes in the aqueous phase, without additional organic solvents. The best results were obtained using ruthenium complex **28** where hydrogen gas with a low CO concentration was produced at 98 °C and constant pressure with a TOF_1h_ (h^−1^)=270 (corresponding to TON=270) and a remarkable TOF_max_value of 719 h^−1^ at a glucose concentration of 0.66 mol L^−1^. The other complexes did not show any exceptional activity. It was observed that hydrogen production increased with acidity of the buffer solution (pH≤0.5). It was noticed that the intermediate product formed during the reaction was formic acid, and this step was proposed as rate‐determining for the overall reaction.^[63]^

The observation that glucose dehydrogenation proceeds via formation of HCOOH as a by‐product was pivotal for the application of highly efficient formic acid dehydrogenation (FADH) catalysts to monosaccharides AD. In 2018, Beller, Li and coworkers described a two‐step process for the direct use of biomass, initially wheat straw, via transformation of its cellulose and hemicellulose components to HCOOH and subsequent H_2_ production by FADH under mild conditions.^[64]^ Initially, the authors assessed the feasibility of the proposed one pot, two step catalytic system in the presence of D‐glucose as model substrate. The hydrolysis‐oxidation first step was obtained in the presence of O_2_ (3 bar) and NaVO_3_ (6 mol %) in 0.7 wt % H_2_SO_4_ solution, followed by acid neutralization. FADH was then achieved in the presence of Ir complex **16**, giving 16 % H_2_ yield (TON=764) from an obtained 51 % yield of FA after 30 h at 90 °C. By addition of dimethyl sulfoxide (DMSO) as co‐solvent (1 %), both FA and H_2_ yields increased under the same reaction conditions, i. e. 74 % (FA) and 54 % (H_2_, TON=2601). Direct use of wheat straw (0.94 g, 18 mmol C‐atoms in polysaccharides) afforded HCOOH in quantitative yields in the presence of NaVO_3_ (4 mol %), DMSO (1 %) 160 °C, 3 h. The resulting mixture was then used for FADH in the presence of proton‐responsive Ir catalysts **35**–**43** (Figure 3).

**Scheme 5:**
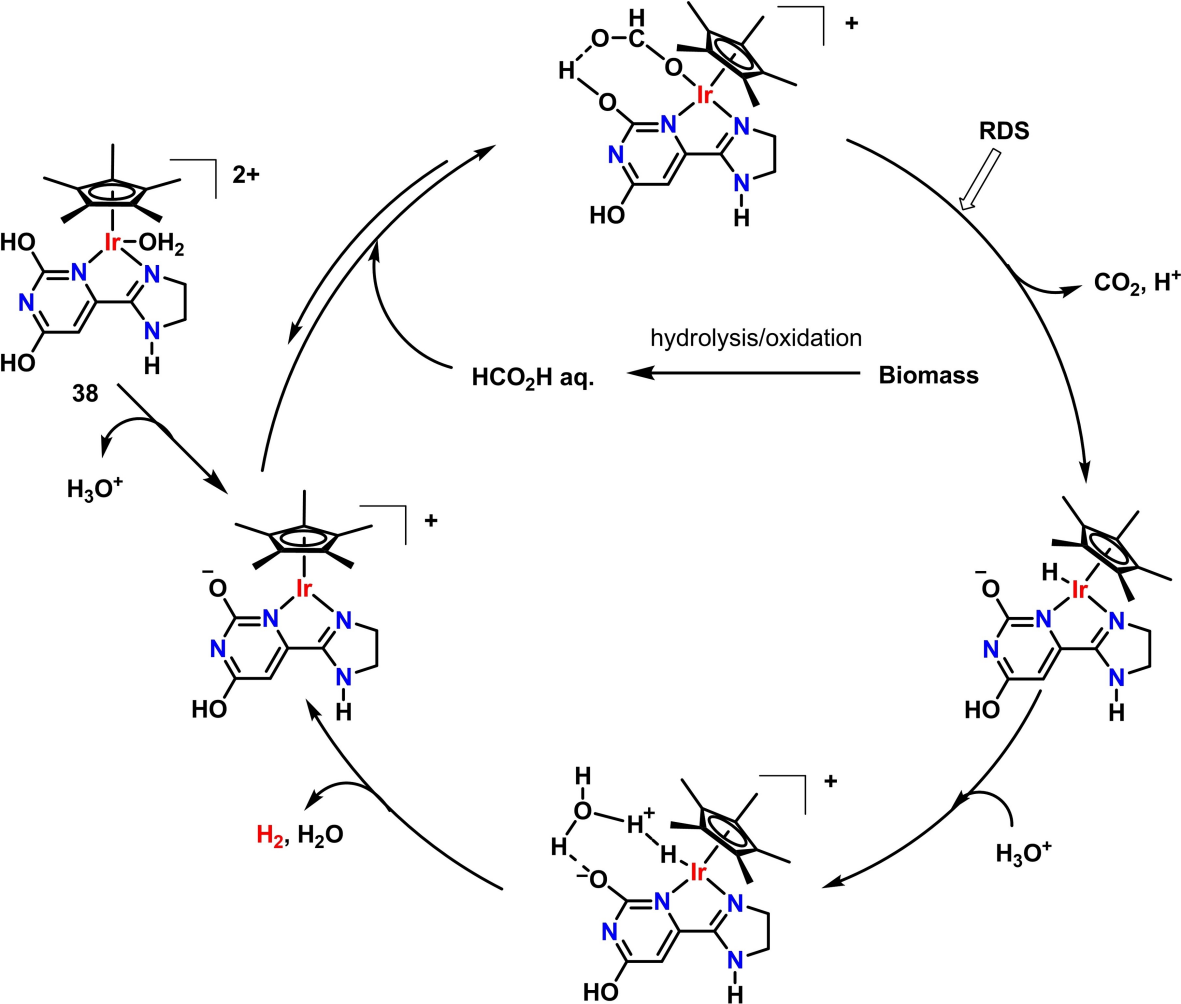
Hydrogen production by FADH reaction catalyzed by **38**, utilizing biomass‐derived HCOOH from the previous hydrolysis‐oxidation step.^[64]^

These complexes feature piano‐stool geometries made of a pentamethylcyclopentadienyl ligand (Cp*) and various *N*,*N*‐ligands, such as 4,4′‐dihydoxy‐2,2′‐bipyridine, 2,2′,6,6′‐tetrahydroxy‐4,4′‐bipyrimidine, 2,4‐dihydoxypyrimidine connected with pyrazole, 2,4‐dihydoxypyrimidine connected with imidazoline and 2,2′‐bis(imidazoline). The coordination sphere around the Ir(III) metal center is completed by either chloride or aquo ligands. The presence of OH substituents or N basic atoms in the N,N ligands backbone allows ligand participation in the FADH mechanism, by reversible proton shuttling,[^65^, ^66^, ^67^, ^68^, ^69^] as shown in Scheme 5 for complex **38**.^[64]^ All tested complexes **35**–**43** showed high H_2_ productivity at different reaction times, as summarized in Table 2, entries 20–31, the highest been reached by complex **38** (87 % yield H_2_, TON=4171, 40 min). Next, catalyst **38** was used for process optimization. Very high catalyst productivity (TON=13627) was achieved in ca. 24 min when catalyst loading was decreased to 69 ppm (Table 2, entry 30) using 2.83 g wheat straw containing 54 mmol C‐atoms of polysaccharide as substrate. Further decrease of catalyst loading (42 ppm) gave a substantial TON increase to ca. 22000, albeit a slightly longer reaction time (1 h) was needed (Table 2, entry 31). Other raw materials such as corn and rice straw, reed, bagasse, bamboo sawdust, cardboard and newspaper were then tested under optimized conditions using **38** (69 ppm), in all cases obtaining high H_2_ productivities with TONs in the range 9796–13587 after 24–30 h. Finally, using wheat straw chippings instead of 200 mesh powder allowed direct utilization of the produced H_2_ in a commercially available PEM fuel cell, giving a stable supply of 100–150 mW for more than 14 h, as an example of practical use on small scale applications. Remarkably, high selectivities toward H_2_ were achieved in most cases upon process optimization, with values of CO and CH_4_ in the evolved gas stream as low as 6 and <2 ppm, respectively.

**Scheme 6:**
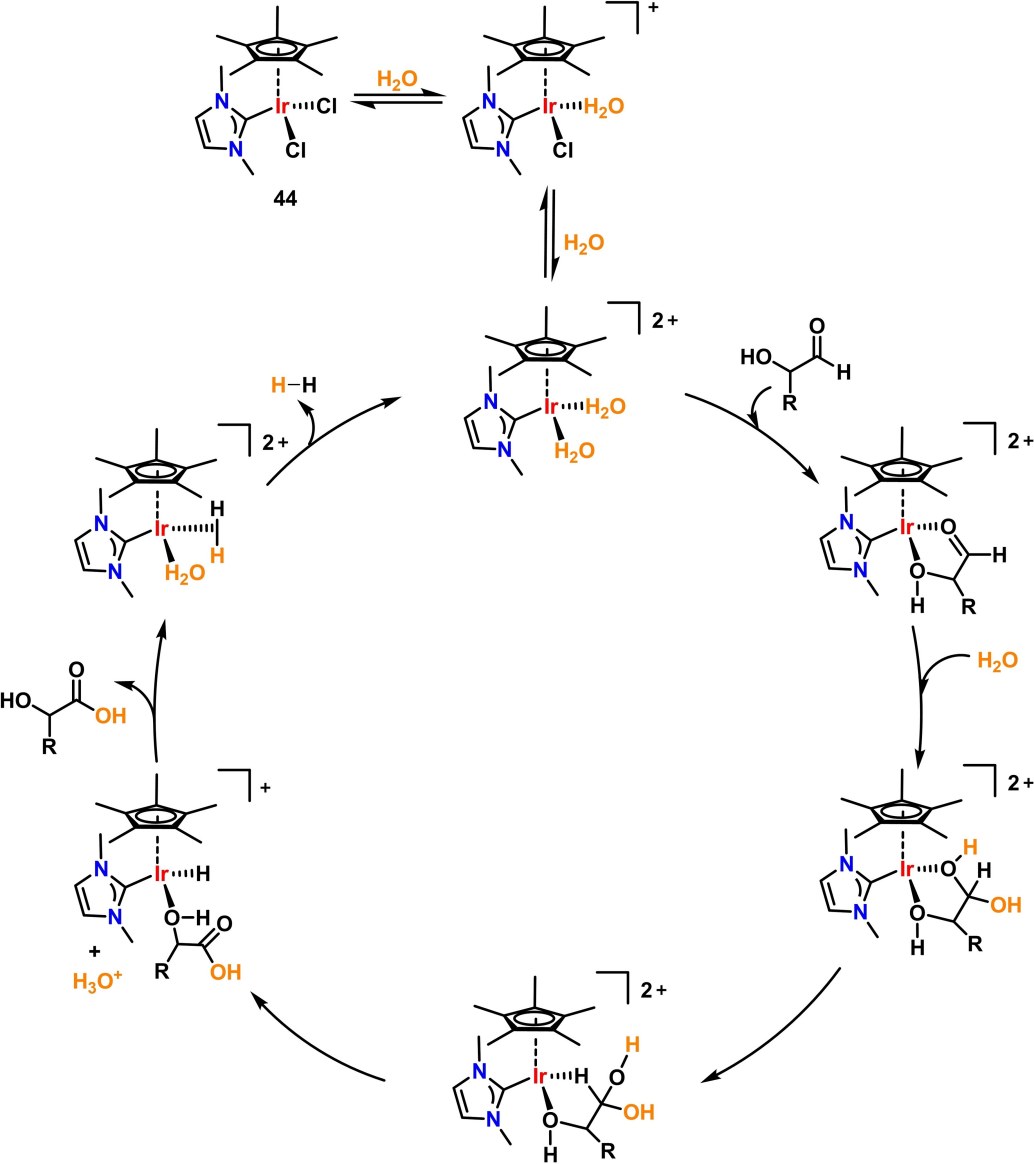
Hydrogen production by glucose AD catalyzed by **44**.^[70]^

The iridium‐NHC complex **44** was used by Garcia, Mata and coworkers. A moderate TON=49 was obtained using complex **44** (2 mol %), D‐glucose and water as a solvent in the presence of H_2_SO_4_ (Table 2, entry 32).^[70]^ Mechanistic investigations led to propose the catalytic cycle shown in Scheme 6. Pre‐catalyst activation involves bis‐substitution of Cl^−^ ligand with H_2_O molecules, giving a bis‐cationic bis(aquo) complex. Labile H_2_O ligands are then exchanged with the incoming substrate in a κ^2^‐coordination mode. The metal‐coordinated substrate then undergoes hydration and protonation steps, to finally release gluconic acid and hydrogen, regenerating the active bis(aquo) species. Notably, this system involves an inner‐sphere mechanism without the assistance of bifunctional proton‐shuttling ligands, which may have be a reason for the lower productivity of compared to **35**–**43**.

Fujita and coworkers used water‐soluble dicationic catalysts **45** and **46**, analogues of **35** except for the *o*‐position of the OH substituents on the bipyridyl ligand and with BF_4_^−^ or CF_3_SO_3_^−^ counteranions, for glucose AD. Catalyst **45** gave hydrogen in 76 % yield (TON=380, Table 2, entry 33). The addition of a strong acid or base was not required during the reaction. Catalyst **46**, bearing tetrafluoroborate anion instead of triflate as in **45**, was tested to investigate the influence of the counteranion. It was observed that with **46** the yield of hydrogen was slightly reduced compared to **45** (71 %, TON=355, entry 35). At higher catalyst loading of **46** (1.0 mol %) H_2_ yield increased to 96 %, albeit with lower TON=95 (Table 2, entry 34). In analogy with Beller and Li′s systems, it was experimentally shown that the AD reaction proceeds involving the OH moiety at 6‐position, however this seems less suitable than the substitution in 4‐position present in **35** that gives higher productivity.^[71]^

Among biomass‐derived polyalcohols, sorbitol is receiving attention as potential AD substrate for its high hydrogen content. Sorbitol is a natural occurring sugar alcohol with a current industrial demand of about 2 Mtonnes/year with uses in chemical, food, textiles, pharmaceutical, health care and cosmetic industries. It is currently produced by hydrogenation of aqueous solutions of starch hydrolysis‐derived D‐glucose using metal‐based reducing catalysts such as Raney Ni.^[72]^ Up to date. three reports describe quantified H_2_ production from sorbitol AD using Ru(II) homogeneous catalysts. Beller and coworkers applied the Ru‐MACHO complex **10**, previously used for glycerol AD, to dehydrogenate sorbitol in diglyme/water (4 : 1), 125 °C, in the presence of KOH (1.5 M) as base. After 1 h of reaction, a TON of 1025 based on measured hydrogen gas was obtained (Table 2, entry 36).^[44]^ In 2023, Daw and coworkers proposed the use of bifunctional pincer‐type [RuCl_2_(PPh_3_)(NNN)] complexes **47**–**49**, differing for the type of substituents in 6‐position on the pincer ligand (OH, OMe, H, respectively). Under optimized conditions (**47**, 0.01 mol %; KOH, 0.5 equiv. to substrate; diglyme/H_2_O 9 : 1; 150 °C, 24 h), TON>35000 was obtained (Table 2, entry 37).

**Scheme 7:**
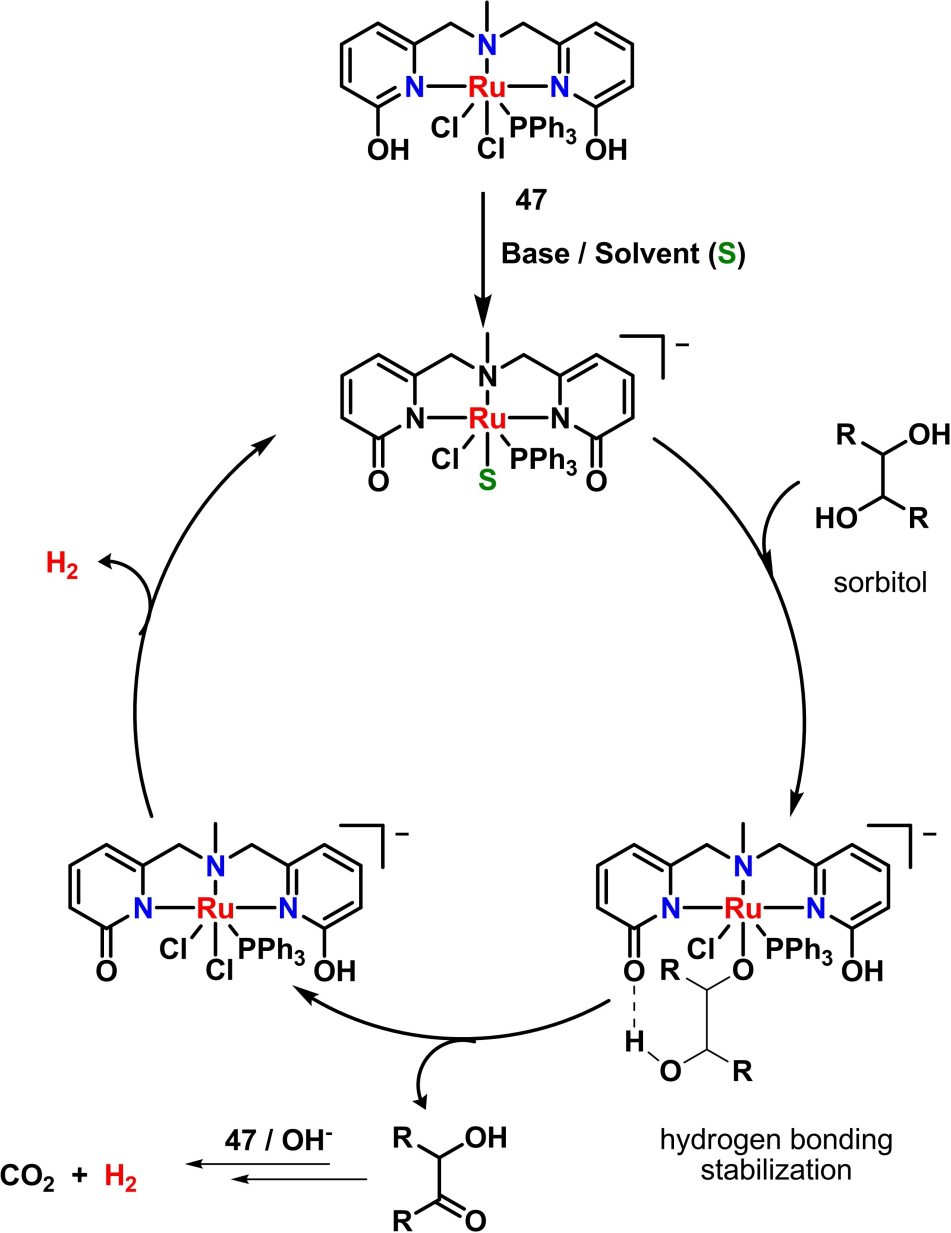
Hydrogen production by sorbitol AD catalyzed by **47**.^[73]^

Interestingly, catalyst recycling for up to seven consecutive runs without significant activity drop was demonstrated, and a total of 1.3 L of H_2_ gas was collected after 192 h of continuous reaction. The presence of protic arms in the ligand scaffold of **47** proved to be essential to achieve high hydrogen production by AD reaction. In detail, the OH‐substituted ligand can induce both a metal–ligand cooperativity pathway and a secondary‐coordination‐sphere hydrogen‐bonding interaction, achieved by appropriate substrate orientation at the active center. The proposed inner‐sphere catalytic mechanism, showing the importance of hydrogen bonding stabilization of the coordinated substrate, is shown in Scheme 7.^[73]^

In the search for catalysts able to tolerate high temperatures and strongly acidic or basic conditions, such as those often needed for polyalcohols AD reactions, Fasolini, Mazzoni and coworkers tested the efficiency of the well‐known commercial and thermally robust Ru bimetallic Shvo catalyst (**50**) and two monometallic analogues, either bearing a NHC ligand (**51**) or an iodide ligand with an imidazolium cationic counterion (**52**) for sorbitol AD, in DMSO/H_2_O (1 : 1), at 150 °C, 4 h, using 2 mol % of catalyst^[74]^ Under these conditions, hydrogen was obtained in ca. 19 %, 2 % and 5 % yields, respectively (Table 2, entries 40–42). Next, complex **50** was tested under the same conditions for other monosaccharides such as fructose and arabinose, and for polyalcohols such as sorbitol and glycerol. The best yield in hydrogen was obtained for arabinose (ca. 15 %), whereas sorbitol and glycerol gave very low yields (0.4 and 1.4 %, respectively). The low hydrogen yields were attributed by the authors to the capacity of Shvo‐type catalyst to consume generated H_2_ to reduce polar double bonds and, by a thorough analysis of the reaction mixtures, they identified hexamethylfurfural (HMF) as one of the reduced by‐products, especially for long time reactions. Based on this observation, 1,4‐benzoquinone (BQ) was added to the reaction to promote catalyst dehydrogenation. BQ is known to behave as a hydrogen acceptor from the reduced catalyst, and in turn this may help in further dehydrogenation of the substrate and favor overall conversion. Although the activity of **50** was only slightly improved during glucose AD tests with 2 mol % BQ (from 19.1 to 23.2 % yield of H_2_), a more evident effect was observed in the presence of 51 and 52, enhancing H_2_ yields from 2.0 % to 6.8 % with **51** and from 4.8 to 17.7 % with **52**, respectively. In general, the use of large excess of BQ had a detrimental effect in catalysis, and this was attributed to the consumption of produced hydrogen in catalyst reduction.

In summary, the best reported results for H_2_ production from pure glucose and fructose homogeneous AD were obtained with the Ir(PNN) pincer complex **14**, a well‐known catalyst for hydrogenation/dehydrogenation reactions, with TONs of ca. 12000 at 95 °C reaction temperature. In the case of sorbitol, a polyalchol that can be obtained from glucose, an outstanding TON>35000 was recorded with the phosphorus‐free Ru(NNN) pincer complex **47**, albeit at higher temperature (150 °C). Another remarkable achievement was demonstrated in the direct use of non‐edible agricultural waste such as wheat straw, via a one‐pot, two‐step process, reaching very high TONs (ca. 20000) in the presence of bifunctional Cp*Ir(NN) complexes, in particular complex **38**, bearing tailored combinations of substituted pyridines, imidazoles, pyrimidines that allow proton‐responsivity. Notably, these highly active homogeneous catalysts work by ligand‐assisted mechanisms, either by MLC‐type amino‐amido ligand reversible activation (in case of PNN pincer complexes) or by proton shuttling bringing about reaction intermediates stabilization (NNN and NN ligands), that is achieved by the presence of proton responsive moieties, typically OH groups in specific positions of the ligand. These geometrical and mechanistic features should be considered in the quest for the next generation of active homogeneous catalysts for sugars AD.

## Summary and Future Perspectives

In this Concept article, we have shown that the use of hydrogen‐rich, largely available and cheap biomass derivatives such as sugars and glycerol, may in future integrate other mature technologies for sustainable hydrogen generation, providing that highly efficient catalysts are discovered and applied for this purpose. Catalytic acceptorless dehydrogenation (AD) reactions allow to extract hydrogen from these platform chemicals by breaking C−H and O−H bonds. Literature data demonstrate the feasibility of this approach, as efficient and selective homogeneous catalysts, mainly based on precious transition metals such as Ru and Ir, can already reach high productivities and selectivity to H_2_ in the evolved gas stream.

The key to obtain suitable catalytic systems is the proper combination of transition metal, functional ligand and geometry. In this view, non‐innocent, MLC‐enabling PNP and PNN pincer‐type ligands, as well as proton‐responsive NN and NNN counterparts were shown to possess both the correct donor properties to stabilize the catalyst metal centers in their low oxidation states and to facilitate key steps of the catalytic mechanism that are crucial for substrate activation, transformation and product(s) release.

Although organometallic complexes synthesis and homogeneous AD catalysis are well‐established concepts and have found practical applications over the last decades, issues still need to be tackled for practical application to biomass polyalcohols dehydrogenation. In our view, the major points to be addressed in future research in this field are:

1. *Catalyst thermal stability*. When compared to heterogeneous catalysts, organometallic and coordination compounds are easier to fine‐tune but in general are less adequate for long‐term, high temperature reaction conditions (>200 °C). The design of novel stabilizing ligands should consider this aspect in the choice of strongly binding donor atoms and robust architectures.
2. *Catalyst recycling*. A common drawback of homogeneous catalysis is catalyst recovery and reuse. Many research groups have addressed this issue for other catalytic applications, developing ligand tethering to surfaces and polymeric materials. When precious metals are used on large scale applications, this issue has an impact on economic sustainability and strategies to immobilize homogeneous catalysts should be considered.
3. *Catalysts water‐tolerance*. Raw substrates from industrial biomass treatment sources come with various amounts of water, that would require energy‐intensive and costly workup to eliminate, decreasing feasibility from an economic point of view. The introduction of polar or charged substituents such as hydroxyl, ammonium or carboxylate groups in the ligand scaffolds can increase catalysts water solubility and may in turn allow for direct use of industrial wet glycerol, for example.
4. *Replacement of precious metals with earth‐abundant counterparts*. In order to further increase the overall economic sustainability, research should be addressed to the replacement of costly transition metals with cheaper counterparts such as 3d earth‐abundant ones. Although this trend in research is already ongoing, and it was successfully demonstrated for other catalytic reactions in recent years,[^75^, ^76^, ^77^, ^78^, ^79^, ^80^, ^81^] it is far from being of simple solution. Transition metals in different groups and rows have different oxidation states, electronic structures, and atomic radii, that in turn rule the geometry, reaction mechanism, and catalytic activity of transition metal complex catalysts. For example, even if they have the same oxidation state, transition metals with different electronic configurations present different ligand field distortion, thus causing geometry choices that may affect the rate‐determining steps of the reaction mechanism (“group effect”). An example is the switch from d^6^ Fe(II)‐PNP to d^7^ Co(II)‐PNP complexes for transfer hydrogenation reactions, where an outer‐sphere mechanism is active for Fe, but not for Co.^[82]^ Next, switching among metals in the same group but different row change the radii of the metal center. This affect the dative binding properties and the coordination geometry, so even in the presence of the same strongly binding ligand, catalyst stability may differ significantly during the (de)hydrogenation runs, for example in switching from Ru(II) to Fe(II).^[83]^ This “row effect” should be carefully considered, and in the design of new ligands researchers should properly evaluate back‐donation ability, electronegativity, dative interaction, and metal center radii, also with the aid of predictive theoretical calculations.^[24]^
5. *Minimize trial‐and‐error catalyst design*. Traditional approaches based on learn‐by‐mistake in catalyst synthesis and testing should be avoided or at least minimized, especially when costly platinum group transition metals are involved. In this regard, established predictive theoretical calculation methods can support the experimental work, by suggesting the most suitable combination of metals and ligands and the appropriate ligand architectures and introduction of functional groups. Calculations at a molecular level can identify the rate limiting step of the reaction mechanisms and propose ways to either modify the catalyst structure to enhance activity to overcome high energy barriers, or the need of co‐catalysts that can assist in promoting specific steps in the reaction pathways.

## Conflict of Interests

The authors declare no conflict of interest.

## Biographical Information

*Sylwia Kostera received her Bachelor Degree (2010), Master Degree (2012) and PhD in Chemistry (2017) from the Adam Mickiewicz University Poznań (Poland). She was Postdoctoral Fellow at the University of Wroclaw (Poland) from 2017 to 2018, then moved to CNR‐ICCOM Florence (Italy) at first as Postdoctoral NAWA Bekker Programme Fellow (2019), and then as Postdoctoral Fellow on various projects since 2020. Her interests include carbon dioxide hydrogenation and hydroboration, precious and earth‐abundant transition metals organometallic chemistry, homogeneous catalysis and study of reaction mechanisms*.

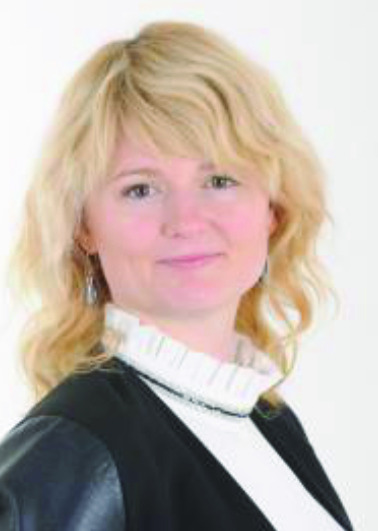

## Biographical Information

*Luca Gonsalvi received his Laurea (M.Sc.) in Chemistry (1994) from the University of Parma (Italy) and Ph.D. in Organometallic Chemistry and Catalysis (1999) from The University of Sheffield (UK). He was Postdoctoral Fellow at Delft University of Technology (NL) from 1999 to 2001, then joined CNR‐ICCOM Florence (Italy) as Researcher, then Senior Researcher (2010–2020) and Research Director (since 2021). He received Habilitations as Full Professor in General and Inorganic Chemistry (2016) and in Industrial Chemistry (2019). His interests include CO_2_ reduction processes, reversible hydrogen storage in LOHCs, precious and earth‐abundant metals organometallic chemistry, homogeneous catalysis in water, study of mechanisms*.